# Linear and Non-linear Analyses of EEG in a Group of ASD Children During Resting State Condition

**DOI:** 10.1007/s10548-023-00976-7

**Published:** 2023-06-18

**Authors:** Brenda Y. Angulo-Ruiz, Francisco J. Ruiz-Martínez, Elena I. Rodríguez-Martínez, Anca Ionescu, David Saldaña, Carlos M. Gómez

**Affiliations:** 1 Human Psychobiology Laboratory, Experimental Psychology Department, University of Seville, C/ Camilo José Cela S/N 41018, Seville, Spain; 2 Département de Psychologie, Université de Montréal, Montréal, Canada; 3 Laboratorio de Diversidad, Cognición y Lenguaje, Departamento de Psicología Evolutiva y de la Educación, University of Seville, C/ Camilo José Cela S/N 41018, Seville, Spain

**Keywords:** Autism spectrum disorder, Multiscale entropy, Power spectral density, Variability, Resting-state

## Abstract

This study analyses the spontaneous electroencephalogram (EEG) brain activity of 14 children diagnosed with Autism Spectrum Disorder (ASD) compared to 18 children with normal development, aged 5–11 years. (i) Power Spectral Density (PSD), (ii) variability across trials (coefficient of variation: CV), and (iii) complexity (multiscale entropy: MSE) of the brain signal analysis were computed on the resting state EEG. PSD (0.5–45 Hz) and CV were averaged over different frequency bands (low-delta, delta, theta, alpha, low-beta, high-beta and gamma). MSE were calculated with a coarse-grained procedure on 67 time scales and divided into fine, medium and coarse scales. In addition, significant neurophysiological variables were correlated with behavioral performance data (*Kaufman Brief Intelligence Test* (KBIT) and *Autism Spectrum Quotient* (AQ)). Results show increased PSD fast frequency bands (high-beta and gamma), higher variability (CV) and lower complexity (MSE) in children with ASD when compared to typically developed children. These results suggest a more variable, less complex and, probably, less adaptive neural networks with less capacity to generate optimal responses in ASD children.

## Introduction

Autism spectrum disorder (ASD) is one of the most prevalent neurodevelopmental disorders in childhood. ASD presents a high degree of heritability (64–91%) (Muhle et al. 2004; Tick et al. 2016). Its etiology is still unknown and although some studies point to genetic alterations, most diagnosed children are non-syndromic. The main symptomatology is characterized by socio-communicative deficits, restricted interests, and stereotyped behaviors (American Psychiatric Association 2013). These clinical signs can be present as early as 12 months, however, diagnosis is currently delayed until the age of 4 years or older (Wang et al. 2013). In the time up to diagnosis, subtle changes in brain function may precede behavioral symptoms that indicate developmental problems. This leads to the need to find potential biomarkers for early detection that would help and support diagnosis (Simon et al. 2017). In this sense, non-invasive techniques for human brain research, such as electroencephalography (EEG), are proposed as an advantageous alternative. The resting state condition require little environmental demands and subject involvement (Wang et al. 2013), which accommodates the clinical characteristics of children diagnosed with ASD, such as their high sensitivity (Simon et al. 2017).

Neurophysiological studies with EEG which have analyzed neural correlates of ASD use linear measures, such as Power Spectral Density (PSD) (Chan et al. 2007; DiStefano et al. 2019; Pierce et al. 2021). The PSD measured as relative power (relationship between bands) or absolute power (degree of electrophysiological activity present in a specific band) has supported the U-shaped curve hypothesis in ASD patients proposed by Wang et al. (2013). The U-shaped curve hypothesis refers to higher power in both low-frequency bands (e.g. delta and theta; Daoust et al. 2004; Chan et al. 2007; Pop-Jordanova et al 2010), and high-frequency bands, (e.g. beta and gamma; Orekhova et al. 2007; Rojas and Wilson 2014), and reduced power in medium-frequency bands (e.g. alpha; Dawson et al. 1995; Chan et al. 2007) for ASD with respect to controls. Nevertheless, to show the complex and variable intrinsic activity underlying neural networks, it becomes necessary to complement PSD analyses with other more specific measures of neural activity. In recent decades, research has increasingly focused on nonlinear measures of EEG, such as brain signal variability and complexity (Takahashi 2013; Garrett et al. 2013; Van Noordt and Willoughby 2021).

Variability is one of the most basic measures of the EEG signal. It estimates the range of values over which the signal oscillates through the standard deviation (SD) or the coefficient of variation (CV) (Garrett et al. 2013; Grady and Garrett 2018). Studies have shown variability increase in normal development (Garrett et al. 2013; Angulo-Ruiz et al. 2021), as well as oscillatory and pathological dependencies (Reviewed in Angulo-Ruiz et al. 2021, 2022). A previous study showed greater relative variability (CV) across trials in the delta band in children with attention deficit hyperactivity disorder (ADHD) compared to normally developing children (Angulo-Ruiz et al. 2022).

Entropy is the main measure of analysis of temporal complexity of brain signals (Kolmogorov 1958; Pincus 1991, 1995; Richman and Moorman 2000). Among all types of entropy (Takahashi 2013), multiscale Entropy (MSE) is one of the most direct measures of intrinsic physiological complexity, allowing the detection of long-range temporal correlations, and reflecting atypical EEG patterns in brain disease (Costa et al. 2002, 2005, Takahashi 2013, 2009; Mizuno et al. 2010; Garrett et al. 2013; Simon et al. 2017; Shen et al. 2021). High and sustained values of complexity at all scales would indicate optimal system performance, while low values would indicate random or a highly predictable signal structure (Papaioannou et al. 2021).

The MSE studies have shown increases during development (McIntosh et al. 2008; Lippe et al. 2009; Garrett et al. 2013; Van Noordt and Willoughby 2021), increases (in fine scales) and decreases (in coarse scales) as a function of the scale range during normal development (Szostakiwskyj et al. 2017; Angulo-Ruiz et al. 2022), as well as decreases with normal aging (Takahashi et al. 2009). In addition, recent studies report partially shared variance between the MSE coarse-graining process and spectral density bands (PSD) in both normal (Bosl et al. 2022) and pathological populations (Angulo-Ruiz et al. 2022).

Studies of clinical populations have often reported abnormal physiological complexity (Takahashi 2013; Shen et al. 2021). Lower complexity compared to typical development has been described in neurodevelopmental disorders (Chu et al. 2017; Angulo-Ruiz et al. 2022). Namely, patterns of lower complexity in ASD children (Bosl et al. 2011; Liu et al. 2017; Kang et al. 2019) and adults (Catarino et al. 2011; Milne et al. 2019) has been reported. However, some studies show increased complexity (Bosl et al. 2017; Takahashi et al. 2016) and/or possible influences of symptom severity (Takahashi et al. 2016; Hadoush et al. 2019). Regardless of the direction of MSE abnormality, these findings could be consistent with inefficient information processing and atypical neural connectivity (McIntosh et al. 2008; Takahashi 2013; O'Reilly et al. 2017).

In this context, our study aims to provide an integrative approach to the analysis of the EEG brain signal of children diagnosed with ASD in comparison to normally developing children. We employ spectral power density (PSD), relative PSD, EEG power variability across trials (CV) and complexity (MSE) analyses. We hypothesized that children with ASD would show lower complexity values compared to the normative group, while showing an increase in EEG variability. For PSD, we expect higher amplitude values in the low (low-delta and delta) and high (high beta and gamma) frequency bands, and lower values in the middle frequencies (theta and alpha) in the ASD group when compared to controls. We expect a possible relationship between PSD and MSE suggesting a partially shared common source of variance. We aim to characterize differences between the ASD and the control groups using these neurophysiological metrics and to possibly provide a tool for better classification and early diagnosis of ASD.

## Methods

### Participants

Thirty-four children participated in this study. The initial sample of the group diagnosed with ASD consisted of 15 children. One child was eliminated after signal processing due to EEG artifacts. The age of the 14 children analyzed was between 5 and 10 years (M = 8.36, SD = 1.22, 1 female). They were recruited from two private centers in Seville, one dedicated to the evaluation and treatment of ASD, and the other from a school with a special education program whose parents reported in detail the diagnosis of ASD. In both cases, the diagnosis was made by expert psychiatrists and clinical psychologists (not directly involved in this study) using CIE-10, DSM-IV, and DSM-V. In each referral private center, the diagnosis was confirmed using Autism Diagnostic Observation Schedule-Generic (*ADOS-G*; Lord et al. 2000).

The typically development or control group was composed of 19 children, who were recruited from different schools in Seville. The parents did not report neurological diseases, signs of epileptic discharge, learning difficulties, or developmental delays in the children. One child was excluded due to technical problems, thus the final sample was composed of 18 children aged between 5 and 11 years (M = 7.89, SD = 1.99, 3 females). There were no significant differences between the groups (control and ASD) neither in age (t(30) = -1.05, p = 0.28, *d* = 0.39), nor in gender (t(30) = -0.79, p = 0.44, *d* = 0.30). Given the absence of significant gender ratio, and the low number of females, the gender factor was excluded for further analysis.

Behavioral tests were conducted to all children who participated in this study (Table 1). Participant attrition and nonresponse (i.e. parents’ incapacity of bringing their child to the assessment day or returning the questionnaires) lead to some missing psychometric data. *SCQ*, and *AQ* were filled by ASD tutors or parents.

**Table 1:** Results of descriptive analysis of behavioral psychometric tests

Group	*SCQ*	*KBIT* (Matrices subtest)	*AQ*
Control		N = 18 (M = 30.22, SD = 5.28)	N = 17 (M = 42.76, SD = 13.70)
ASD	N = 11 (M = 21.55, SD = 7.16)	N = 13 (M = 28, SD = 8.77)	N = 14 (M = 87.64, SD = 22.50)

The clinical group was assessed with the *Social Communication Questionnaire* (*SCQ*-lifetime; Rutter et al. 2003), a screening questionnaire for parents based on the *Autism Diagnostic Interview-Revised* (*ADI-R*; Lord et al. 1994; Berument et al. 1999; Yau et al. 2016) to determine the presence of the disorder. A cut-off point of 15 points was established to confirm the presence of the disorder (Mercader and Miranda 2016). The total score of the questionnaire showed that 10 subjects of the clinical group scored above the cut-off point and only one scored below. Three parents did not return the questionnaire. However, the clinical diagnosis prevailed and all the 14 ASD referred children were included for further analysis.

The parent version of the *Autism Spectrum Quotient* (*AQ*; Baron-Cohen and Wheelwright 2004) was used in both groups to measure the expression of autistic characteristics and to corroborate the diagnoses made by the clinical centers. A cut-off point of 76 was established to confirm the presence of ASD symptoms (Auyeung et al. 2008). Seventeen children from the control group (one missing) and four from the ASD group scored below the cut-off point (76). While 10 children from the ASD group scored above 76. However, the clinical diagnosis prevailed and the 14 children with ASD referred were included for further analysis. Total *AQ* scores showed significant differences between groups (t(29) = -6.84, p < 0.001, *d* = 2.41), with ASD group presenting a higher mean score (M = 87.64, SD = 22.50) than control group (M = 42.76, SD = 13.70).

In addition, the Matrices subtest of the *Kaufman Brief Intelligence Test* (*KBIT*; Kaufman and Kaufman 2004) was employed to measure nonverbal cognitive skills. As this measure does not require participants to give a verbal response, it is considered the most appropriate given the age and diagnosis of the participants. Given the heterogeneity and inequality among cognitive profiles typical of this clinical population, *KBIT* scores were only used to characterize the sample and not to exclude individuals due to low IQ (Jarrold and Brock 2004; Ruiz-Martínez et al. 2020). Direct *KBIT* scores showed no differences between groups (t(29) = 0.813, p = 0.427, *d* = 0.31).

Following the guidelines of the Declaration of Helsinki, written informed consent was obtained from the parents. They received both written information and an explanation from the researcher about the protocol to be followed (prior to the experimental day, see Experimental session). The aim of the protocol was to make parents aware of the experimental requirements of the children, and thus minimize anxiety in the session. The experimental protocol was approved by the biomedical research ethics committee of the autonomous community of Andalucía.

### Experimental Session

Before the experimental session each parent was provided with a "storyboard" explaining the order of the experimental session from beginning to end. A photograph of each researcher and recording setting was also included. Parents of children with ASD were requested to practice at home with a swimming cap for 20–30 min while watching a movie without sound.

Spontaneous EEG activity was obtained in an eyes-open condition for 3 min in a soundproof room decorated with child-friendly elements to establish a suitable environment for the children. The subjects were instructed to look at a cross on the screen and to remain in a relaxed position, blinking as little as possible. Nineteen electrodes according to the international 10–20 system were used (ELECTROCAP) (Fp1, Fp2, F3, F4, C3, C4, P3, P4, O1, O2, F7, F8, T3, T4, T5, T6, Cz, Fz, Pz), including a ground electrode. An average reference was used and due to the high somatosensory reactivity of the ASD group, ocular and mastoid electrodes were avoided. To record blink artifacts, the signal amplitude of the frontal electrodes was used. Data were recorded at a gain of 20,000 and at a sampling rate of 1024 Hz in direct current using an analog-to-digital acquisition and analysis system (ANT) without any filtering. Impedance was kept below 10 Kilo-Ohms.

### Data Analysis

The EEGLAB toolbox (Delorme and Makeig 2004) and the Matlab R2021b software package were employed for EEG data analysis. A notch filter of 47–53 Hz was applied (EEGLAB function: *eegfiltnew*) and average reference was used. An Artifact Subspace Reconstruction (ASR) algorithm (EEGLAB Function: *clean rawdata*) was used to correct portions of data with a standard deviation exceeding by 20 times the one of the calibration data. To correct for eye, blinks, muscle and other movement artefacts, an independent component analysis (ICA) was performed in EEGLAB (Function: *pop_runica*). Components carrying artefacts were tracked by ICLabel classification (Pion-Tonachini et al. 2019) and removed. All epochs (2000 ms) with ± 120 μV of the 19 channels were rejected (EEGLAB Function *eegthresh*). Subjects had a range of selected epoch between 14 and 90 for analysis (M = 73.69, SD = 19.62), and a range of 14 to 18 accepted components (M = 15.66, SD = 1.06). There was a significant difference between groups in the number of accepted epochs (t(30) = 2.93, p = 0.016, *d* = 0.99, mean control = 81.72, SD = 10.55; mean ASD = 63.36, SD = 23.84), but no group differences were found for the number of selected components. There was not a significant correlations of the number of epochs with age.

### LPSD and Relative PSD Analysis

Mean (M) and standard deviation (SD) of the logarithm of the absolute PSD (LPSD) across trials were calculated with the EEGLAB *spectopo* function. This uses the Matlab *pwelch* function while applying a Hamming window. LPSD was calculated in 2-s windows with 2048 sampling points and a sampling rate of 1024 Hz. The frequency resolution of the LPSD spectrum was 0.5 Hz. LPSD was averaged over different frequency ranges: low-delta (0.5–1 Hz), delta (1.5–3 Hz), theta (4–7.5 Hz), alpha (8.5–13 Hz), low-beta (13.5–20 Hz), high-beta (20.5–30 Hz) and gamma (30.5–45 Hz).

To obtain the absolute PSD (LPSD = 10 * Log (PSD)), needed for computing the relative PSD, the logarithm of the LPSD was removed (PSD = e^LPSD/10^). The relative PSD spectrum was obtained from absolute PSD. The relative PSD of each electrode and subject was calculated according to the following formula:

$${X}_{(fi)}=\frac{{PSD}_{(fi)}}{{\sum }_{i=0.5}^{45}{PSD}_{(fi)}}*100$$

where X(fi) is the relative PSD for a given frequency. PSD (fi) is the absolute PSD for a given frequency. Σ*PSD*(*fi*) is the sum of the absolute PSD at all frequencies considered (0.5–45 Hz in 0.5 Hz steps).

### Variability Across Trials

To obtain a measure of the variability of PSD across trials, the mean of the absolute PSD (M) in a given frequency band and its corresponding trial-by-trial standard deviation (SD) were divided to obtain the CV across trials (CV = SD/M).

### Multiscale Entropy Analysis

Using the Matlab function *MultiscaleSampleEntropy* (Malik 2022), we calculate Multiscale Entropy (MSE) for all channels. Malik (2022) based his function on the MSE method of Costa et al. (2005), which calculates sample entropy (S_E_; Richman and Moorman 2000) at multiple time scales using a coarse-grained procedure. MSE measures signal complexity (Garrett et al. 2013) by dividing the EEG signal into non-overlapping windows of a different number of samples. Each time scale is defined by averaging the different neighboring points (p) of the original time series (of length τ). Thus, the repetition frequency of m-point versus m + 1-point patterns is evaluated. It is necessary to define a similarity limit (r) to delimit the tolerance range in which individual neighboring points are considered similar (k). This similarity limit is normalized by the standard deviation (SD) of the EEG k < r × SD (Malik 2022). *S*_*E*_ is then calculated for each time scale (Malik 2022):

$$SE=log\frac{ {p}^{m}(r)}{{p}^{(m+1)}(r)}$$

Therefore, MSE is calculated using a coarse-grained process that filters out high frequencies with increasing scales (Kosciessa et al. 2020; Bosl et al. 2022). This process has recently been related to power spectral analysis using Haar wavelet approximations (Bosl et al. 2022). Thus, MSE low scales would contain all frequencies found in the EEG signal, whereas high scales would only contain low frequencies (Bosl et al. 2022).

Following recommendations provided in previous studies on EEG signal complexity (Richman and Moorman 2000; McIntosh et al. 2008; Miskovic et al. 2016; Kloosterman et al. 2019; Kosciessa et al. 2020), the parameters used in this study were m = 2 and r = 0.5. MSE was calculated for 67 time scales. The latter corresponding to 30 time points obtained by collapsing 67 consecutive sampled points (0.97656 ms x 67scales = 65.4 ms), in each 2 s trial. A complete description of the number of points, sample periods and frequencies included in each MSE scale are described in supplementary table 1.

High complexity would be defined by high values of S_E_ indicating a low repetition of patterns of length “m” over “m + 1” and an optimally functioning system (Papaioannou et al. 2021). Low complexity would indicate random or high similarity of patterns indicating information poverty (McIntosh et al. 2008; Garrett et al. 2013).

### Statistical Analysis

#### ANOVA

**Fig. 1:**
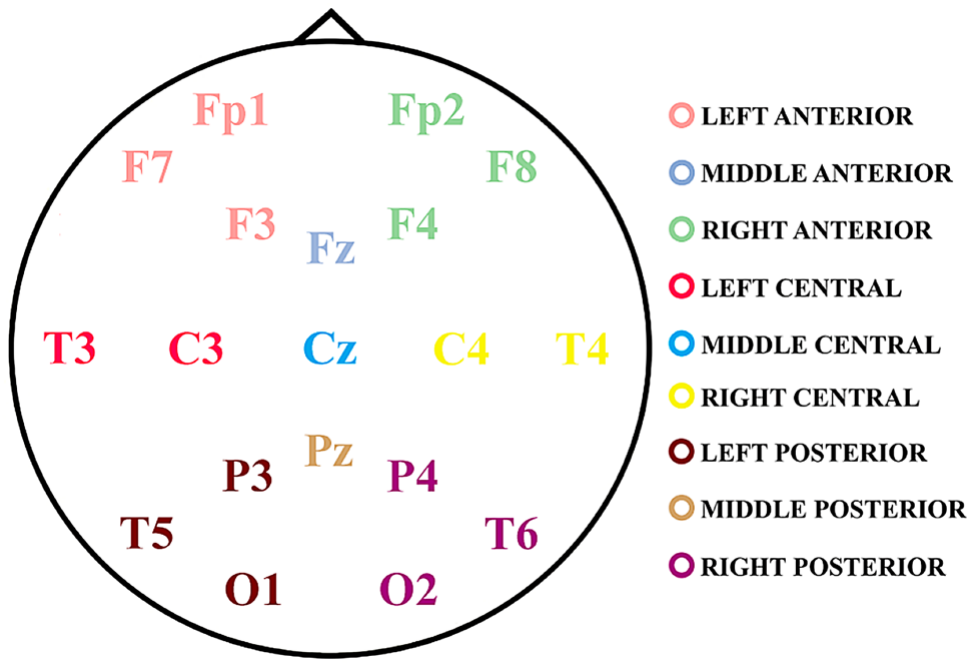
Localization and collapse of electrodes by regions**.** The colors indicate the nine defined scalp areas for electrodes collapse

To reduce the dimensionality of the data, neighboring electrode values of each calculated parameter (LPSD, CV, relative PSD and MSE) were collapsed into 9 areas (Fig. 1). Also, PSD (logarithm and relative) and CV were collapsed into different frequency bands (see Table 2 for the EEG frequency ranges used). The MSE, in turn was organized based on three different types of scales, corresponding to the temporal sampling of scales proposed by Szostakiwskyj et al. (2017). Thus, fine scales were considered from scale 1 (0.98 ms and 2048 time points per trial) to scale 25 (24.4 ms and 81 time points), medium scale from scale 26 (25.4 ms and 78 time points) to scale 45 (43.9 ms and 45 time points) and, finally, coarse scale from scale 46 (44.9 ms and 44 time points) to scale 67 (65.4 ms and 30 time points), see supplementary table 1 for parameters of the different scales.

**Table 2:** Significant results were obtained for the logarithm of the PSD (LPSD)

BANDS	RM-ANOVA	t-tests
Low-delta (0.5–1 Hz)	Within-subjects: Laterality x group p = 0.006 F(1.81, 52.44) = 6.14, ηp^2^ = 0.175, power = 0.846	Left-medial: t(30) = − 3.02, p = 0.008, *d* = 1.15 Medial-right: t(30) = 3.02, p = 0.008, *d* = 1.06
Delta (1.5–3 Hz)	Within-subjects: Laterality x group p < 0.001 F(1.93, 55.82) = 9.75, ηp^2^ = 0.252, power = 0.974	Left-medial: t(30) = − 3.89, p < 0.001, *d* = 1.37 Medial-right: t(30) = 3.89, p < 0.001, *d* = 1.36
Theta (4–7.5 Hz)	Within-subjects: Laterality x group p < 0.001 F(1.76, 50.97) = 13.08, ηp^2^ = 0.311, power = 0.992 Anterior–posterior x group p = 0.055 F(1.94, 56.32) = 4.54, ηp^2^ = 0.135, power = 0.742	Medial: t(30) = 4.65, p < 0.001, *d* = 1.66 Central: t(30) = 3.16, p = 0.011, *d* = 1.14 Posterior: t(30) = 2.60, p = 0.021, *d* = 0.95
Alpha (8.5–13 Hz)	Within-subjects: Laterality x group p = 0.015 F(1.74, 50.53) = 4.85, ηp^2^ = 0.143, power = 0.737	Left-medial: t(30) = -2.53, p = 0.025, *d* = 0.89 Medial-right: t(30) = 2.75, p = 0.025, *d* = 0.97
Low-beta (13.5–20 Hz)	Within-subjects: Laterality x group p = 0.002 F(1.99, 57.91) = 7.85, ηp^2^ = 0.213, power = 0.942 Anterior–posterior x group p = 0.055 F(1.80, 52.31) = 5.19, ηp^2^ = 0.152, power = 0.778	Left-medial: t(30) = -2.97, p = 0.014, *d* = 1.10 Medial-right: t(30) = 3.90, p = 0.001, *d* = 1.38 Anterior–posterior: t(30) = -3.23, p = 0.009, *d* = 1.14
High-beta (20.5–30 Hz)	Between-subjects Group p = 0.042 F(1, 29) = 7.16, ηp^2^ = 0.198, power = 0.734 Within-subjects: Laterality x group p = 0.005 F(1.96, 56.89) = 6.22, ηp^2^ = 0.177, power = 0.873	Right: t(30) = -3.39, p = 0.006, *d* = 1.18
Gamma (30.5–45 Hz)	Between-subjects: Group p = 0.041 F(1,29) = 8.81, ηp^2^ = 0.233, power = 818 Within-subjects: Laterality x group p = 0.005 F(1.99, 57.97) = 6.03, ηp^2^ = 0.172, power = 0.867	Right: t(30) = -3.30, p = 0.007, *d* = 1.15

All p-values of the RM-ANOVA and t-test were corrected by FDR correction. The RM-ANOVA factors used were: group of subjects, anterior–posterior, laterality, and covariate (age in days). The RM-ANOVA was computed independently for each frequency band. The effect size for RM-ANOVA was computed by eta partial squared (ηp^2^), and for posthoc t-tests by means of Cohen’s *d*

Data were analyzed using Repeated Measures ANOVAs (RM-ANOVA) with age (in days) as a covariate in the Statistical Package for the Social Sciences 25 (SPSS). RM-ANOVAs size effects were directly computed in SPSS using eta partial squared (ηp^2^). In addition, false discovery rates (FDR) were used to correct for multiple comparisons (Benjamini and Hochberg 1995). Only significant results for the factors considered in the RM-ANOVA that included the group factor and survived FDR correction were reported and discussed, given the main purpose of the report.

For mean PSD (LPSD and relative PSD) and CV, RM-ANOVA analysis was performed independently for the seven frequency bands (low-delta, delta, theta, theta, alpha, low-beta, high-beta and gamma). Age in days was used as a covariate, group (control and ASD) as a between-subjects factor, and anteroposterior (anterior, central and posterior) and lateral (left, medial and right) areas as within-subjects factors.

For MSE, the between-subjects factor was group (control and ASD), the covariate was age (in days) and the within-subjects factors were scale type (fine, medium and coarse), anteroposterior areas (anterior, central and posterior) and lateral areas (left, medial and right).

For posthoc analysis, Student's t-tests were computed with Cohen's d as the effect size metrics (Cohen 1988). All p-values were corrected for multiple comparisons using the FDR (Benjamini and Hochberg 1995).

#### Spearman Correlation Between MSE and Relative PSD

In order to verify the relationship between the spectral power (PSD) and the MSE (Bosl et al. 2022), we performed a Spearman correlation between relative PSD vs. MSE, with age (in days) when controlling correlation by age.

#### EEG Metrics vs. Behavioral Correlational Analysis

For the behavioral vs. EEG correlation analyses, only group significant EEG metrics were used. Psychometric tests used for correlation were those completed by both groups (control and ASD), such as the *KBIT* (as a measure of non-verbal general intelligence) and the *AQ* (to characterize subjects with ASD). Spearman partial correlations controlling for age were performed for all subjects without distinguishing between groups, given the small sample size. P-values were corrected by FDR (Benjamini and Hochberg 1995).

## Results

**Fig. 2:**
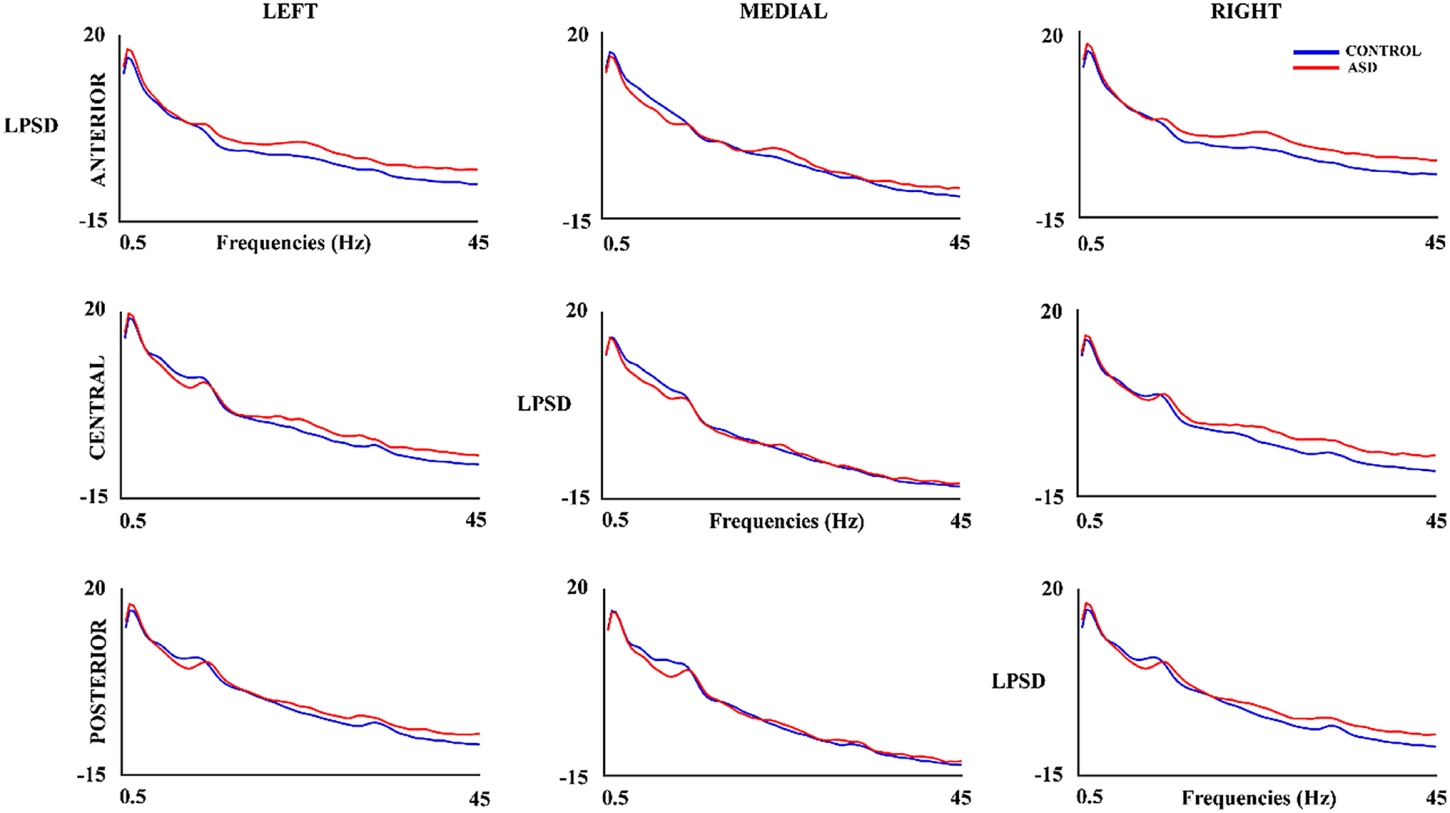
Logarithm of the Power Spectral Density (LPSD) in control and ASD subjects

Figure 2 shows the LPSD in all considered areas for the control and ASD groups. The RM-ANOVA (Table 2 and in summary Table 6) shows main and interaction group effects (with FDR correction) of high-beta and gamma bands. Mean of high-beta LPSD of ASD (M = − 3.52, SD = 2.18) was higher than the LPSD of the control group (M = − 4.99, SD = − 1.23). A similar pattern was obtained for the gamma band (ASD: M = − 6.95, SD = 2.31; control: M = − 8.60, SD = − 1.56). All bands showed laterality x group interaction effects. T-test comparison (Table 2) between left and medial areas showed higher mean to ASD group in low-delta (M = 0.93, SD = 1.60), delta (M = 0.80, SD = 1.18), alpha (M = 0.82, SD = 0.92), and low-beta bands (M = 2.05, SD = 1.30) than control group (low-delta: M = − 0.52, SD = 1.11; delta: M = − 0.65, SD = 0.92; alpha: M = 0.074, SD = 0.753; low-beta: M = 0.97, SD = 0.48). The difference between medial and right areas of the mean of LPSD was higher in control group in low-delta (M = 0.168, SD = 1.25), delta (M = 0.48, SD = 0.91), alpha (M = − 0.07, SD = 0.934), and low-beta bands (M = − 1.02, SD = 0.94) than ASD group (low-delta: M = − 1.37, SD = 1.63; delta: M = − 1.01, SD = 1.25; alpha: M = − 1.02, SD = 1.03; low-beta: M = − 2.37, SD = 1.02). The posthoc analyzed in the theta band showed higher mean in medial areas (p < 0.001) in control group (M = 8.23, SD = 1.25) than ASD group (M = 6.20, SD = 1.19). In high-beta and gamma bands the mean of LPSD in right areas (high-beta: p = 0.006; gamma: p = 0.007) was higher in ASD group (high-beta: M = − 2.09, SD = 2.19; gamma: M = − 5.08, SD = 2.52) than control group (high-beta: M = − 4.30, SD = 1.50; gamma: M = − 7.59, SD = 1.79). Only theta and low-beta bands showed an interaction effects between the group with the anterior–posterior factor. The central (p = 0.011) and posterior (p = 0.021) areas showed higher mean of LPSD in control group (central: M = 7.07, SD = 1.35; posterior: M = 8.22, SD = 1.52) than ASD group (central: M = 5.67, SD = 1.09; posterior: M = 6.97, SD = 1.08) in theta band. The low-beta band showed higher differences between anterior and posterior areas in the ASD group (M = 0.37, SD = 1.08) with respect to control group (M = − 0.79, SD = 0.951).

RM-ANOVA for relative PSD (Table 3 and in summary Table 6) with FDR correction showed main group effect in theta band where the highest relative PSD values were for the control group (M = 2.33, SD = 0.44) compared to ASD group (M = 1.79, SD = 0.57). No group significant interaction effects were obtained for any frequency band.

**Table 3:** Significant results obtained in the RM-ANOVA of the relative PSD with factors: group of subjects, anterior–posterior and laterality, and age in days like covariate

BANDS	Relative PSD
Theta (4–7.5 Hz)	Between-subjects: Group p = 0.029 F(1, 29) = 9.65, ηp^2^ = 0.250, power = 0.851

The RM-ANOVA was computed independently for each frequency bands. For the calculation of relative PSD, the logarithm was eliminated. P-value with FDR correction

The RM-ANOVA of the CV across trials (Table 4 and in summary Table 6) with FDR correction showed group main effects in the theta and alpha bands. In the theta band, the CV was higher in the ASD group (M = 1.85, SD = 0.108) compared to the control group (M = 1.79, SD = 0.030). Similarly, the CV in the alpha band was higher in the ASD group (M = 1.94, SD = 0.099) than in the control group (M = 1.83, SD = 0.060).

**Table 4:** Significant results obtained in the RM-ANOVA of the Coefficient of variation (CV) across trials with factors: group of subjects, anterior–posterior and laterality, and age in days like covariate

BANDS	CV
Theta (4–7.5 Hz)	Between-subjects: Group p = 0.044 F(1, 29) = 7.08, ηp^2^ = 0.196, power = 0.730
Alpha (8.5–13 Hz)	Between-subjects: Group p = 0.008 F(1, 29) = 12.79, ηp^2^ = 0.306, power = 0.933

The RM-ANOVA was computed independently for each frequency bands. For the calculation of CV across trials, the logarithm of the LPSD was eliminated. All the p-values with FDR correction

**Fig. 3:**
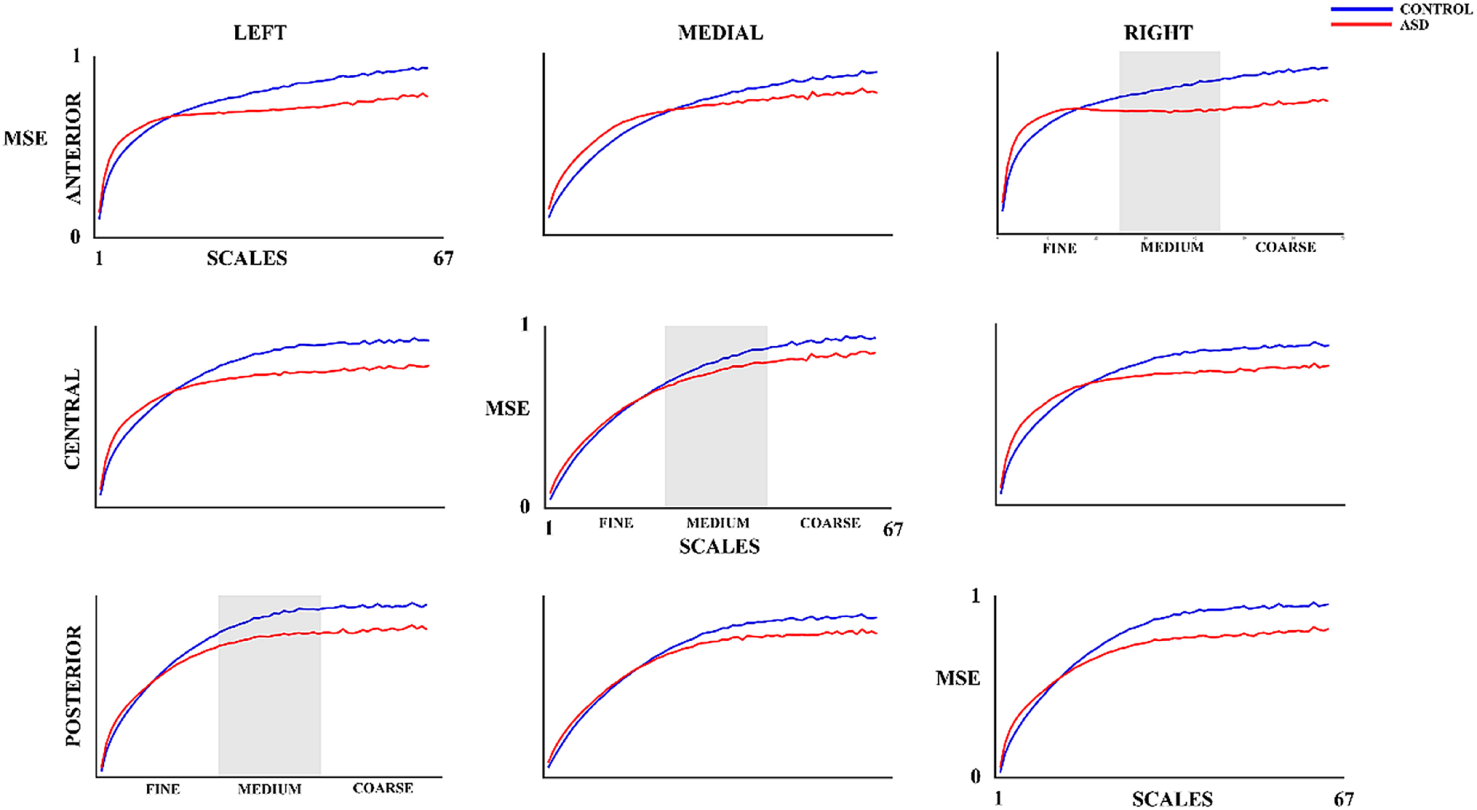
Multiscale Entropy (MSE) for 67 scales in control and ASD subjects. The medium scale is highlighted to differentiate the three types of scales

Figure 3 shows the MSE results for the control and ASD groups. The MSE increases as the scale increases for both groups. However, as the MSE values of the control group exhibit a constant rise, the MSE of the ASD group shows a rise in fine scales and a plateau from the medium scales upwards. The RM-ANOVA of the MSE (Table 5 and in summary Table 6) shows a main group effect with the control group presenting a higher mean MSE (M = 0.79, SD = 0.059) than the ASD group (M = 0.74, SD = 0.082). In addition, group differences in the medium scales (t(30) = 2.44, p = 0.030, *d* = 0.78) and coarse scales (t(30) = 3.49, p = 0.005, *d* = 1.19) are found based on the interaction effect results (with FDR correction). The MSE in both medium and coarse scales was higher in control group (medium: M = 0.86, SD = 0.077; coarse: M = 0.92, SD = 0.062) than ASD group (medium: M = 0.79, SD = 0.099; coarse: M = 0.82, SD = 0.101). In addition, significant differences were obtained in the interaction of the effects of laterality x group factors. These differences were due to a higher MSE in controls (left: M = 0.79, SD = 0.060; right: M = 0.78, SD = 0.064) than in ASD group (left: M = 0.73, SD = 0.084; right: M = 0.72, SD = 0.077) for both left (t(30) = 2.48, p = 0.030, *d* = 0.82) and right (t(30) = 2.45, p = 0.030, *d* = 0.85) lateral areas.

**Table 5:** Significant results obtained in the RM-ANOVA of MSE with factors: group of subjects (controls and ASD), scales (fine, medium, and coarse), anterior–posterior, and laterality, and age in days as a covariate. All results with FDR correction

Between-subjects Group p = 0.022 F(1, 29) = 5.83, ηp^2^ = 0.167, power = 0.646 Within-subjects: Scales x group p = 0.001 F(1.26, 36.47) = 10.99, ηp^2^ = 0.275, power = 0.938 Laterality x group p = 0.019 F(1.88, 54.48) = 4.38, ηp^2^ = 0.131, power = 0.715

**Table 6:** Summary of RM-ANOVA results for all calculated parameters

Parameters	Between-subjects	Within-subjects (interaction with the group)
LPSD	High-beta (ASD > C) Gamma (ASD > C)	Low-delta: -Diff. Left-Medial (ASD > C) -Diff. Medial-Right (C > ASD) Delta: -Diff. Left-Medial (ASD > C) -Diff. Medial-Right (C > ASD) Theta: -Medial (C > ASD) -Central (C > ASD) -Posterior (C > ASD) Alpha: -Diff. Left-Medial (ASD > C) -Diff. Medial-Right (C > ASD) Low-beta: -Diff. Left-Medial (ASD > C) -Diff. Medial-Right (C > ASD) -Diff. Anterior–Posterior (ASD > C) High-beta: -Right (ASD > C) Gamma: -Right (ASD > C)
Relative PSD	Theta (C > ASD)	
CV	Theta (ASD > C) Alpha (ASD > C)	
MSE	C > ASD	Medium (C > ASD) Coarse (C > ASD) Left (C > ASD) Right (C > ASD)

In brackets: control > ASD (C > ASD) or ASD > control (ASD > C). *Diff* differences

Table 6 shows a summary of the RM-ANOVA results.

**Fig. 4:**
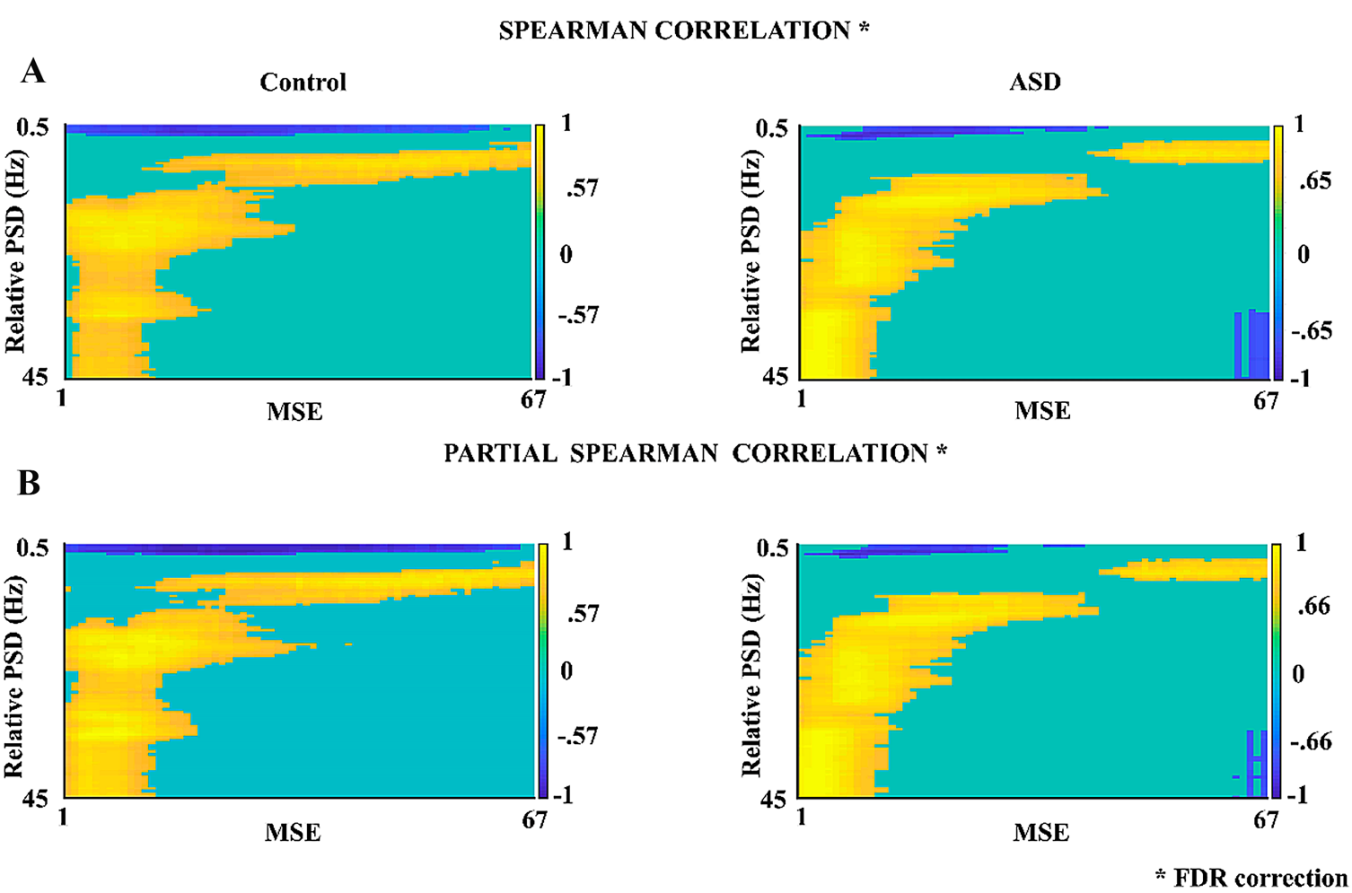
**A** Spearman Correlation between MSE (67 scales) and relative PSD (0.5–45 Hz) in control and ASD subjects. **B** Partial Spearman Correlation controlled for age (in days) for both groups of subjects. Significant cutoff values for each correlation (with FDR correction) are indicated in the graphs. In this correlation, we calculated up to a total of 67 scales to observe the effect of correlation with increasing scales

Figure 4 shows the results of the Spearman and partial correlation (controlling for age) between relative PSD (0.5–45 Hz) and MSE (scales 1–67), both with FDR correction. Significant positive and negative correlations are observed both for the control group (positive with cutoff for Rho > 0.57, p < 0.015; negative with cutoff for Rho > -0.57, p < 0.016) and for the ASD group (positive with cutoff for Rho > 0.65, p < 0.014 and negative with cutoff for Rho > -0.66, p < 0.013).

The obtained significant positive correlations were observed between frequencies higher than 5 Hz and all MSE scales. Significant negative correlations were also found between low frequencies (< 2.5 Hz) and MSE of all the scales considered, in both groups analyzed. The calculated partial correlations do not show a maturation effect in either group.

Regarding the correlation between EEG neurophysiological metrics (summary in Table 6) and behavioral tests (*AQ* and *KBIT*), the results lost significance after correction for multiple comparisons.

## Discussion

We analyzed different neurophysiological measures of power, variability and complexity of the EEG in resting state to observe possible alterations between a group of children diagnosed with ASD compared to a normodevelopment group. Previous studies using correlational analyses of MSE vs. age have shown an increase in complexity with age as a product of better adaptability to cognitive demands and possible reconfigurations of the functional network produced by a more variable state (McIntosh et al. 2008, 2010; Van Noordt and Willoughby 2021). A strong dependence of age for the PSD has also been previously described (Reviewed in Rodriguez-Martinez et al. 2021). As such, the present study includes age as a covariate in all the statistical analyses to compensate for any change in the EEG parameters due to age. Our results show an increase in PSD (LPSD and relative) in the theta band in the control group and an increase in high frequency bands (high-beta and gamma in LPSD) in the ASD group, as well as higher variability (CV) across trials, and lower complexity (MSE) compared to typically developed children.

PSD studies have been shown to be a reliable biomarker of EEG maturation, with decreases in the absolute values of brain rhythms throughout development, as well as decreases in low frequency bands and increases in high frequency bands in the relative PSD (Gasser et al. 1988; Segalowitz et al. 2010; Miskovic et al. 2015; Rodríguez-Martínez et al. 2020, 2021). This would be related with the selection of stable neuronal connections during the synaptic pruning process, producing an increase in the efficiency of neuronal transmission throughout development (Whitford et al. 2007).

Our results showed differences between groups in the theta band with higher LPSD and relative PSD values in the control group and, in the high-beta and gamma bands of LPSD with greater power among the ASD group with respect to control. Regarding the alpha band, several studies reported increased or decreased alpha power in children with ASD, high-functioning ASD, or ASD risk populations (Lazarev et al. 2009; Tierney et al. 2012; Carter et al. 2018; DiStefano et al. 2019; Pierce et al. 2021). Our results in this band focus mainly on laterality x group interactions, where the ASD group presents higher levels of LPSD in left vs. medial lateral areas and lower levels in medial vs. right areas. However, our results support the possible biomarker role of gamma band activity as suggested by Rojas and Wilson (2014). These authors suggest gamma activity as a reliable endophenotype of ASD given the weakening of perceptual and cognitive functions in the autism population. However, the direction of amplitude change of gamma activity remains controversial, given that certain studies found an increase of gamma power in ASD (Orekhova et al. 2007, 2008; Machado et al. 2015), while others found a decrease in power (Sheikhani et al. 2009, 2012; Maxwell et al. 2015; Van Hecke et al. 2015). Present results would support the increase in gamma amplitude in ASD. These results would partially support the U-shaped hypothesis of PSD suggested by Wang et al. (2013) in which children with ASD present higher PSD values in low and high-frequency bands compared to a control group, as well as lower values in mid-frequency bands such as the theta band.

EEG signal variability was analyzed based on the absolute PSD CV across trials metric. In this study, the CV, which corresponds to a measure of PSD variability across trials (Angulo-Ruiz et al. 2021), showed an increase in ASD for theta and alpha bands compared to the control group, suggesting greater variability of spontaneous EEG in the ASD group across trials. Similarly, increased neural signal variability has been shown in other clinical populations (Castellanos et al. 2009; Angulo-Ruiz et al. 2022). As CV solely corresponds to a first-order measure of variability, complexity measurements related to multiplicity of EEG patterns at different temporal scales, such as MSE, are more suitable for assessing abnormal physiological EEG signals (Takahashi 2013; Chu et al. 2017; Papaioannou et al. 2021; Shen et al. 2021).

The ASD group shows a slowdown in the increase of MSE with scales from the middle scales onwards yielding lower entropy values from these scales with respect to controls, implying a defective functional system (McIntosh et al. 2008; Papaioannou et al. 2021; Van Noordt and Willoughby 2021). Both increase and decrease of complexity in ASD subjects have been shown in the literature. Bosl et al. (2017) in EEG, and Takahashi et al. (2016) in Magnetoencephalography (MEG) have reported increases in MSE in the ASD group. Lower EEG complexity was also found in this clinical group (Bosl et al. 2011; Chu et al. 2017; Liu et al. 2017; Kang et al. 2019). This reduction of complexity in MSE in ASD would imply a reduced adaptability, given the reduction of possible patterns in the EEG, possibly related to a less variable repertoire of neural network dynamics that would impair behavioral adaptation (McIntosh et al. 2008; Takahashi 2013; O'Reilly et al. 2017).

The relationship between connectivity and complexity has been shown previously and explained in terms of neural connections (Friston 1996; Sporns et al. 2000; Takahashi 2013). Therefore, the large-scale integration efficiency resulting from synchronization between small and large neuronal populations evolving in different frequency ranges could be reflected in the physiological complexity of the signal (Takahashi 2013). Lower entropy values in ASD suggests abnormal information processing and connectivity, mainly in long-range neural interactions, given the relationship between coarse scales and low frequency bands (Szostakiwskyj et al. 2017; Bosl et al. 2022). The latter argument is supported by the relationship of long range connections with low EEG frequencies, and of short range connections with high EEG frequencies (Lea-Carnall et al. 2016). In this sense, abnormal complexity could support the theory of local overconnectivity and long-range underconnectivity, which is central to the underlying pathology of ASD (Belmonte et al. 2004; Courchesne and Pierce 2005; Geschwind and Levitt 2007; Wass 2011) and other neurodevelopmental disorders such as attention deficit hyperactivity disorder (Clarke et al. 2008). Moreover, impaired connectivity has been found in ASD, such as decrease of electrophysiological connectivity and long-range connections (Rippon et al. 2007; Chan et al. 2011; Duffy and Als 2012; Takahashi 2013; Yuk et al. 2020). Our results support local overconnectivity and long-range underconnectivity hypothesis for ASD due to the decrease of MSE coarse scales (decrease in long range connectivity), and by the increase of highbeta and gamma PSD (increase of local connectivity). Although, it must be indicated that this hypothesis remains as controversial, despite a higher support for the long-range underconnectivity hypothesis (O’Reilly et al. 2017).

The MSE decrease in coarse scales suggests lower complexity and greater signal predictability in ASD with respect to controls. The relationship between PSD and MSE shown in the present report and others (McIntosh et al. 2008, 2010; Szostakiwskyj et al. 2017; Bosl et al. 2022; Angulo-Ruiz et al. 2022) support the statement about the relationship between high frequencies and fine scales, as well as between low frequencies and coarse scales, both in the normo-developmental and clinical group, even when this relationship is controlled by age. In this regard, the relation of scales and frequency bands in our study with a sampling rate of 1024 Hz and 67 scales would include in the coarse scales (coarsest scale ≤ 7.64 Hz bands): the low-delta, delta, theta bands; medium scales: alpha and low-beta bands (in addition to low frequencies); and in the fine scales, high-beta and gamma bands (in addition to low and medium frequencies) (Bosl et al. 2022). The strong relationship between the fine scales of the MSE and high-frequency bands would be related to local connectivity and/or local processing. The same is implied to the relationship between the coarse scales and low-frequency bands with global connectivity or long-range interactions (Szostakiwskyj et al. 2017; Van Noordt and Willoughby 2021; Bosl et al. 2022). Present results of correlations between MSE and PSD suggest that there is some shared variance for these two metrics, in both ASD and controls.

The correlation of the EEG metrics with the AQ and KBIT test were not significant after correction for multiple comparisons. Other studies have shown a relationship between EEG complexity (Bosl et al. 2011), spectral power (Carter et al. 2018) and autistic traits and cognitive brain capacity (McIntosh et al. 2008). However, given the heterogeneity of the disorder, it is possible that there are multiple individual neural profiles in ASD and thus multiple pathways to its features and symptoms (Milne et al. 2019). The small sample size does not permit analysis of differential correlational patterns in both groups of subjects.

This work has some limitations that could influence the reported results. The low sample size and the limited age range could explain the disparity found with other developmental studies, in which specific developmental patterns of power (Segalowitz et al. 2010; Rodríguez-Martínez et al. 2020; Angulo-Ruiz et al. 2021) and complexity (Szostakiwskyj et al. 2017; Angulo-Ruiz et al. 2022) are shown in healthy subjects. Likewise, it is important to include more women in the studies and to analyze the possible existence of gender dependencies, as Huberty et al. (2021) and Cragg et al. (2011) found in their spectral power studies. Statistical power is another considerable limitation given our sample size and low range age, however our results show a minimum power of 0.64 with a large effect size (ηp^2^ = 0.167), and a maximum power of 0.99 with a large effect size (ηp^2^ = 0.311) (Cárdenas-Castro and Arancibia-Martini 2014). Although the statistical power and effect sizes were generally high in this report, the results described should be taken with caution due to the sample size. In this sense the loss of significance in some metrics could be due to the effect of a low sample size. Therefore, it is important to consider incorporating a higher number of participants for future experiments. Finally, we suggest a longer period than 3-min resting state recording period for a longer epoch segmentation and a more reliable estimation of MSE.

## Conclusions

The higher LPSD values of the ASD group in high-frequency bands, higher variability (CV) across trials, and lower EEG complexity (MSE) would indicate abnormal functioning at the neural and functional level in ASD, which is in line with previous literature on ASD and other neurodevelopmental disorders (Chu et al. 2017; Angulo-Ruiz et al. 2022).
